# Spontaneous grain refinement effect of rare earth zinc alloy anodes enables stable zinc batteries

**DOI:** 10.1093/nsr/nwae205

**Published:** 2024-06-13

**Authors:** Manjing Chen, Yuxiang Gong, Yunxiang Zhao, Yexin Song, Yan Tang, Zhiyuan Zeng, Shuquan Liang, Peng Zhou, Bingan Lu, Xiaotan Zhang, Jiang Zhou

**Affiliations:** School of Materials Science & Engineering, Hunan Provincial Key Laboratory of Electronic Packaging and Advanced Functional Materials, Central South University, Changsha 410083, China; School of Materials Science & Engineering, Hunan Provincial Key Laboratory of Electronic Packaging and Advanced Functional Materials, Central South University, Changsha 410083, China; Institute of Materials Research, Tsinghua Shenzhen International Graduate School, Tsinghua University, Shenzhen 518055, China; School of Materials Science & Engineering, Hunan Provincial Key Laboratory of Electronic Packaging and Advanced Functional Materials, Central South University, Changsha 410083, China; School of Materials Science & Engineering, Hunan Provincial Key Laboratory of Electronic Packaging and Advanced Functional Materials, Central South University, Changsha 410083, China; Department of Materials Science and Engineering, City University of Hong Kong, Hong Kong 999077, China; School of Materials Science & Engineering, Hunan Provincial Key Laboratory of Electronic Packaging and Advanced Functional Materials, Central South University, Changsha 410083, China; Hunan Provincial Key Defense Laboratory of High Temperature Wear-Resisting Materials and Preparation Technology, Hunan University of Science and Technology, Xiangtan 411201, China; School of Physics and Electronics, Hunan University, Changsha 410082, China; School of Materials Science & Engineering, Hunan Provincial Key Laboratory of Electronic Packaging and Advanced Functional Materials, Central South University, Changsha 410083, China; School of Chemistry and Materials Science, University of Science and Technology of China, Hefei 230026, China; Suzhou Institute for Advanced Research, University of Science and Technology of China, Suzhou 215123, China; School of Materials Science & Engineering, Hunan Provincial Key Laboratory of Electronic Packaging and Advanced Functional Materials, Central South University, Changsha 410083, China

**Keywords:** grain refinement, rare earth, GB stability, zinc metal batteries

## Abstract

Irreversible interfacial reactions at the anodes pose a significant challenge to the long-term stability and lifespan of zinc (Zn) metal batteries, impeding their practical application as energy storage devices. The plating and stripping behavior of Zn ions on polycrystalline surfaces is inherently influenced by the microscopic structure of Zn anodes, a comprehensive understanding of which is crucial but often overlooked. Herein, commercial Zn foils were remodeled through the incorporation of cerium (Ce) elements via the ‘pinning effect’ during the electrodeposition process. By leveraging the electron-donating effect of Ce atoms segregated at grain boundaries (GBs), the electronic configuration of Zn is restructured to increase active sites for Zn nucleation. This facilitates continuous nucleation throughout the growth stage, leading to a high-rate instantaneous-progressive composite nucleation model that achieves a spatially uniform distribution of Zn nuclei and induces spontaneous grain refinement. Moreover, the incorporation of Ce elements elevates the site energy of GBs, mitigating detrimental parasitic reactions by enhancing the GB stability. Consequently, the remodeled ZnCe electrode exhibits highly reversible Zn plating/stripping with an accumulated capacity of up to 4.0 Ah cm^−2^ in a Zn symmetric cell over 4000 h without short-circuit behavior. Notably, a ∼0.4 Ah Zn||NH_4_V_4_O_10_ pouch cell runs over 110 cycles with 83% capacity retention with the high-areal-loading cathode (≈20 mg cm^−2^). This refining-grains strategy offers new insights into designing dendrite-free metal anodes in rechargeable batteries.

## Teaser

Incorporating cerium into zinc metal anodes refines the grains and increases the density of grain boundaries to suppress zinc dendrites and side reactions by enhancing electron transfer and nucleation sites.

## INTRODUCTION

Zinc metal batteries (ZMBs) have been identified as promising candidates for large-scale energy storage due to their high theoretical capacity (820 mAh g^−1^), low redox potential (−0.76 V vs. standard hydrogen electrode), abundance of zinc (Zn) reserves, and environmental friendliness [1,2]. Despite these advantages, non-dense nucleation and growth processes remain significant challenges for ZMBs [3]. Zn ions tend to deposit in regions with a stronger electric field, leading to the formation of Zn dendrites. This phenomenon results in capacity decay, short-circuiting, and safety risks [4,5]. The attainment of stable and dendrite-free Zn anodes with extended lifespan is crucial for the practical application and commercialization of ZMBs [6].

Several methodologies have been proposed to tackle the challenges of dendrite growth and side reactions, including interface modifications [7], electrolyte optimization [8], three-dimensional (3D) substrate design [9], and anode alloying [10]. However, it is noteworthy that interface modification and electrolyte optimization may lead to an increase in the interfacial impedance or polarization [11,12]. The 3D substrate design of the Zn metal anode is susceptible to collapse during prolonged Zn plating/stripping, and its large specific surface area may exacerbate hydrogen evolution and corrosion reactions [13]. In contrast, Zn anode alloying is regarded as a promising method for fundamentally optimizing Zn metal anodes without the introduction of additional materials [14,15]. The inclusion of other metal elements can regulate nucleation sites and deposition dynamics of Zn. Various strategies for alloying the Zn anode are currently explored, including: (i) introducing alloying elements with a high affinity for Zn to increase nucleation sites and induce uniform Zn deposition [16,17]; (ii) incorporating metals with a high Gibbs free energy of hydrogen adsorption into Zn metal to impede the hydrogen evolution and corrosion reactions [18,19]; (iii) constructing specialized 3D alloy structures to provide large specific areas and mitigate local current density [10,20]. It is crucial to emphasize that the aforementioned modification methods primarily focus on the macroscopic electrode-electrolyte interface. However, metal ions diffuse and transport through grain boundaries (GBs) [21], suggesting that adjusting the GB characteristics of the Zn electrode could fundamentally enhance the transport dynamics of Zn ions [22]. Recent studies have shown that grain refinement can yield abundant GBs and nucleation sites, facilitating the uniform nucleation and growth of Zn deposits. Additionally, the reduced grain size unexpectedly suppresses the ‘tip effect’ of Zn dendrite growth, enhancing the reversibility of metal electrodes [23].

In recent years, rare earth elements have experienced growing utilization in the energy field, driven by their abundant reserves, cost-effectiveness, and distinctive physicochemical properties [8,24,25]. In Zn-based batteries, rare earths are employed to form cerium (Ce)-based oxide films on the Zn anode surface, which effectively mitigate GB corrosion [24]. Additionally, the introduction of Ce ions, lanthanum (La) ions, and other rare earth ions into the electrolyte helps regulate Zn-ion deposition sites by forming a dynamic electrostatic shielding layer [8,25]. Furthermore, rare earth elements contribute to grain refinement in alloys, a technique used to enhance the mechanical properties of metals by reducing the grain size. Known for their ability to form stable compounds with other elements, rare earth elements enable effective control over alloy grain size. Their incorporation as nucleation sites in alloys facilitates the generation of new grains, reducing the size of grains within the crystal. Moreover, rare earth elements function as inhibitors to restrict grain growth within the alloy. This dual functionality prevents excessive grain enlargement, maintaining the desired grain size and enhancing overall mechanical properties [26,27]. Inspired by these insights, the integration of rare earth elements to Zn metal electrodes not only fortifies their mechanical properties to stabilize Zn metal but also increases the density of GBs to accelerate the transport kinetics of Zn ions [22].

In this study, ZnCe electrodes were fabricated through electrodeposition, utilizing the ‘pinning effect’ to incorporate Ce elements at GBs. The incorporation of Ce at GBs effectively reshapes the microstructure of Zn metal anodes, inducing spontaneous grain refinement. Electron backscatter diffraction (EBSD) characterization reveals a significant reduction in grain size for the deposition layer of the ZnCe electrode (∼1 μm) compared to the bare Zn electrode (∼3 μm). This fine-grained microstructure enables precise and compact deposition, allowing meticulous control over electrode performance. Moreover, the segregation of rare earth elements at GBs elevates the site energy (E_site_) of GBs, contributing to the inhibition of corrosion reactions. As expected, the ZnCe||ZnCe symmetrical cells demonstrate an extended cycle life of over 4000 h at 2 mA cm^−2^/2 mAh cm^−2^. Additionally, ZnCe||NH_4_V_4_O_10_ (NVO) full cells achieve 6000 cycles with 96% capacity retention. The strategies employed for regulating grain size in ZnCe metal electrodes hold promise for enhancing the reversibility of other metal electrodes.

## RESULTS

**Figure 1.**
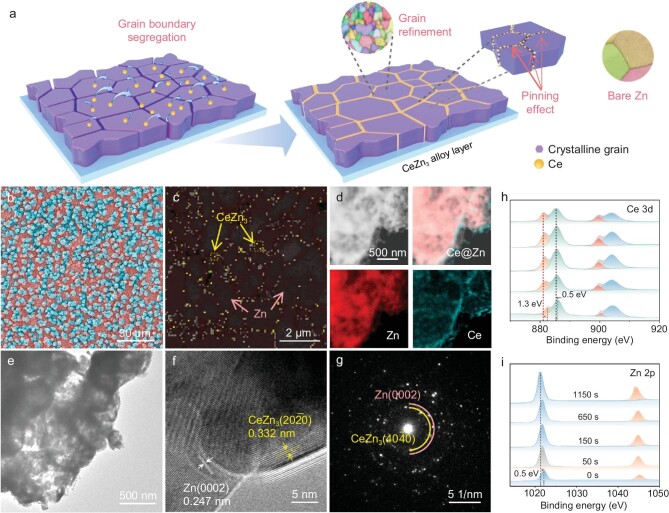
Preparation illustration and structural characterization of the ZnCe electrodes. (a) Schematic illustration of the preparation mechanism for URAL. (b) SEM image of the URAL. The surface protrusions are colored blue. (c) Phase color distribution chart in EBSD. (d) The corresponding elemental mapping images. (e) TEM image, (f) HRTEM image, and (g) SAED pattern of the CeZn_3_@Zn. Depth etching profiles from the XPS analysis of (h) Ce element and (i) Zn element in the ZnCe electrodes.

Figure 1a illustrates the preparation process of the ZnCe electrodes, wherein an ultra-fine rare earth alloy layer (URAL) was constructed on the Zn foil surface through a facile underpotential co-deposition method. The Zener pinning effect in the Zn electrode induces spontaneous grain refinement by impeding GB migration and suppressing crystal growth. This phenomenon is supported by EBSD characterization, revealing a significant reduction in grain size to the sub-micron level, confirming microstructural remodeling. The electrochemical interplay at the electrolyte-electrode interface is affected by the macroscopic surface's topography, underscoring the importance of considering its morphology. Notably, the EBSD phase color diagram (Fig. 1c) analysis identifies Ce elements distributed at the GBs of Zn metal, further confirmed as CeZn_3_ alloy-precipitated phases by X-ray diffraction characterization (XRD; Fig. S1). Under optimal conditions, the ZnCe alloy layer exhibits a distinctive morphology with protrusions conforming to a normal distribution, averaging ∼2.9 μm, evenly dispersed across the Zn foil surface (Figs S2 and S3, and Fig. 1b). A URAL thickness of ∼5 μm is revealed from the cross-sectional scanning electron microscope (SEM) images to distinguish it from the deposition layer in subsequent analyses (Fig. S4). High-angle annular dark-field scanning transmission electron microscopy (HAADF-STEM) characterization, accompanied by corresponding mapping images, demonstrates the uniform distribution of Zn and Ce elements within the CeZn_3_ alloy phase (Fig. 1d). Transmission electron microscopy (TEM) was further conducted to analyze the crystal structure of URAL (Fig. 1e). The subsequent high-resolution transmission electron microscope (HRTEM) image (Fig. 1f) exhibits ordered lattice fringes with spacings of 0.332 and 0.247 nm, corresponding to the CeZn_3_(2$0{{\overline{2}}}$0) and Zn(0002) planes, respectively. Meanwhile, the corresponding selected area electron diffraction (SAED) pattern shows clear diffraction rings of CeZn_3_(40${\mathrm{\overline{4}}}$0) and Zn(0002) crystal planes (Fig. 1g). The obtained results, consistent with the crystal structure determined by inductively coupled plasma optical emission spectrometer (ICP-OES; Table S1), confirm the coexistence of metallic Zn and CeZn_3_ alloy in URAL.

The chemical structure of the URAL was analyzed using X-ray photoelectron spectroscopy (XPS) in conjunction with valence and geometric depth distributions (Fig. 1h and i, and Fig. S5). Prior to etching, peaks indexed to the Ce element are initially observed at binding energies of 881.1 and 885.3 eV in the outermost surface region [28]. Subsequently, with an increase in the etching depth, a slight downshift of 1.3 and 0.5 eV in the binding energy of Ce is observed, which suggests a decrease in the valence state of the Ce element within the alloy layer. Additionally, the binding energy of Zn undergoes a transition from 1022.1 to 1021.6 eV during the etching process, indicating a slight oxidation state of both Zn and Ce elements in the surface region [15].

**Figure 2.**
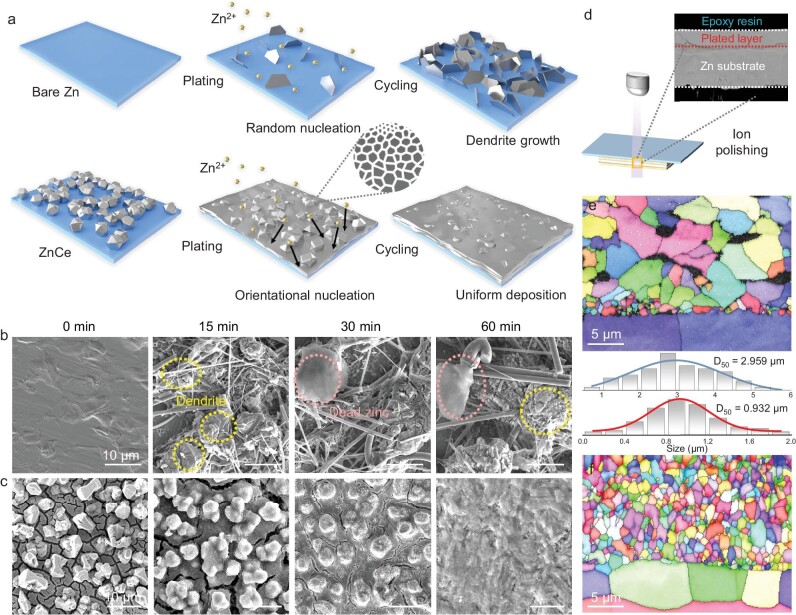
Deposition morphology evolution of bare Zn and ZnCe electrodes. (a) Model of the Zn-ion deposition process on the bare Zn and ZnCe electrodes. *Ex-situ* SEM images of (b) bare Zn and (c) ZnCe electrodes after plating 0, 15, 30, and 60 min at a current density of 2 mA cm^−2^, respectively. (d) Argon ion polishing method used for preparing EBSD samples. EBSD inverse pole mappings of (e) bare Zn and (f) ZnCe electrodes after plating at 2 mAh cm^−2^ and the corresponding grain size distribution curves.

The Ce-based URAL plays a pivotal role in optimizing Zn deposition behavior owing to its robust zincophilicity and pronounced super-hydrophilicity. Contact angle tests (Fig. S6) indicate that the ZnCe electrode exhibits a highly hydrophilic nature with a contact angle of 9.1° when subjected to a 3 M ZnSO_4_ electrolyte, whereas the bare Zn electrode displays a contact angle of 103.0°. This improved hydrophilicity is attributed to the high-specific surface areas and specific alloy components facilitated by the homogeneous micro-nano structure on the URAL surface [19]. Additionally, the adsorption energy between a Zn atom and CeZn_3_ substrate was calculated (Fig. S7), which exhibits significantly lower adsorption energy compared to a Zn atom on a bare Zn substrate. This suggests a preference for surface Zn ions to adsorb and deposit onto the hetero-nucleation sites of CeZn_3_. Figure 2a shows a schematic illustration of the Zn deposition process on the bare Zn and ZnCe electrodes, respectively. On the bare Zn surface, the dendrite-like Zn distributes randomly in certain regions and gradually proliferates, showing irregular and random Zn growth (Fig. 2b) [8]. In contrast, the unique micro-nano structure on the URAL surface contributes to the uniform distribution of Zn-ion flux and directs flat Zn deposition [10]. This phenomenon is further confirmed by SEM, where Zn ions preferentially deposit at the gaps of protrusions, achieving a uniform and dense deposition surface after 1 h of plating (Fig. 2c). In addition, the corresponding stripping process is studied in Fig. S8.

To investigate Zn deposition behavior at the grain scale on the electrode surface, EBSD was employed. Typically, deposited Zn is too thin to be polished, addressed by embedding Zn samples in epoxy resin and ion polishing from the side (Fig. 2d). EBSD images along vertical planes (Fig. 2e and f) reveal that based on the epitaxial growth mechanism, Zn deposits on the ZnCe electrode surface have an average grain size of 0.932 μm, while the Zn grains deposited on bare Zn surface are initially relatively large and then grow much larger, presenting an average grain size of 2.959 μm over three times that of deposited Zn on ZnCe electrode. Moreover, some regions in the EBSD image of the bare Zn electrode are not identified, indicating corrosion reaction and the generation of by-products in these areas. Therefore, the Ce-based URAL not only continuously induces homogeneous Zn deposition with a smaller grain size but also effectively suppresses side reactions.

**Figure 3.**
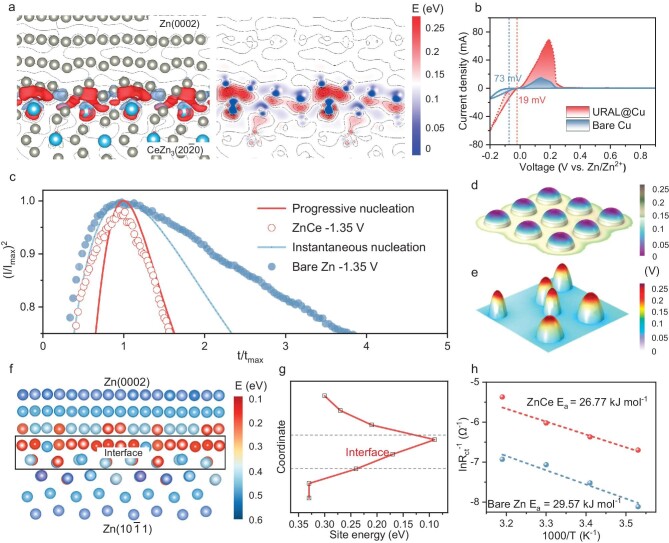
Calculations and experiments on the kinetics of ZnCe electrodes. (a) Distribution of deformation charge density at the phase interface between Zn and CeZn_3_. Red and blue colors indicate accumulation and depletion of the electrons, respectively. (b) Typical CV curves for Zn||Cu half cells at 1 mV s^−1^. (c) Current-time transient graphs of bare Zn and ZnCe electrodes at different potentials, compared with the classical model of progressive nucleation and instantaneous nucleation. (d, e) COMSOL simulations of Zn deposition at 0.2 V for 1 min. (f) E_site_ of Zn atoms in Zn(0002), Zn(10${\mathrm{\overline{1}}}$1), and their interface. (g) Line chart of E_site_. (h) Arrhenius curves and comparison of activation energies.

The function of the URAL in regulating Zn nucleation and growth kinetics was further elucidated through a combination of theoretical calculations and experimental analyses. First-principles calculations were conducted to analyze the charge density between Ce and Zn atoms at the interface of Zn metal phase and CeZn_3_ alloy phase (Fig. 3a) [22]. Owing to the greater electronegativity of the Zn atom in comparison to the Ce atom, electrons are transferred from Ce to the Zn, resulting in an increased electron density surrounding Zn. This result suggests that the higher electron density provides numerous nucleation sites for Zn deposition, fastening Zn ions deposition kinetics. This is further supported by the cyclic voltammetry (CV) curves of URAL@Cu||Zn and bare Cu||Zn half-cells, as depicted in Fig. 3b, the significantly larger closed area and higher current intensity indicate the presence of abundant active nucleation sites on URAL for Zn [19]. As shown in Fig. S9, at different current densities of 0.5, 1.0, 1.5, 2.0, and 2.5 mA cm^−2^, the nucleation overpotentials of the ZnCe electrode are significantly lower than those of the bare Zn electrode, indicating a more effective and favorable nucleation process for the former, minimizing the energy barrier. This reduction in overpotential is crucial for controlling deposition kinetics and ensuring stable and uniform growth of the deposit on the electrode surface (Fig. S10).

Current transients obtained by the chronoamperometry method provide insight into the nucleation mechanism on ZnCe electrodes (Fig. S11). Unlike the conventional instantaneous nucleation model (where nucleation density first increases rapidly and then decreases) and progressive nucleation model (where nucleation density progressively increases and then saturates) [29], the Zn nucleation on the ZnCe electrode follows a mixed nucleation mechanism, that is, instantaneous nucleation in the early stage followed by a saturation like in the scenario of progressive nucleation. The abundant nucleation sites and lower nucleation barrier on the ZnCe electrode lead to the initial rapid activation of numerous nuclei. Maintaining nucleation site activity is crucial for nucleus incubation, while the abundant GBs in URAL facilitate high Zn ion conductivity, ensuring continuous Zn ions contact through solid pathways and thus keeping nucleation sites active for gradual nucleus incubation, aligning with the subsequent progressive nucleation model (Fig. 3c). The utilization of a high-speed instantaneous-progressive nucleation composite model facilitates a meticulous control over the deposition process, resulting in a compact and defect-free electrodeposited layer. In contrast, Zn ions on bare Zn electrodes consistently undergo instantaneous nucleation, with the grains continuing to grow over time, resulting in irregular and relatively large grains. Finite element simulations using COMSOL Multiphysics were performed to further investigate the post-nucleation growth process. On the bare Zn electrode, block-like Zn deposits are randomly scattered on bare Zn, resulting in numerous Zn ions accumulating at the top of the deposits, eventually forming Zn dendrites. Conversely, a uniform charge is distributed on the ZnCe surface to effectively reduce the local current density and contribute to flat and dense Zn deposition (Fig. 3d and e).

First-principles calculations were utilized to evaluate the reactivity of Zn atoms within grains and at GBs. Specifically, Zn(10${\mathrm{\overline{1}}}$1) and Zn(0002) crystal planes are selected, and the reactivity is quantitatively assessed through the E_site_. E_site_ is defined as the energy difference between E_0_ and E_vacancy_, where E_0_ represents the total energy of the defect-free model, and E_vacancy_ represents the energy of the model with a Zn vacancy [22]. Figure 3f and g illustrate the similarity in reactivity between Zn(10${\mathrm{\overline{1}}}$1) and Zn(0002) crystal planes, while the E_site_ at the GBs markedly exceeds that within the crystal. This observation implies a preference for Zn-electrolyte reactions at GBs, indicating a propensity for Zn ions to diffuse along GBs. The URAL has abundant GBs to improve the transport kinetics of Zn ions by modulating Zn plating/stripping behaviors. This results in a reduced charge transfer resistance compared to bare Zn, as evidenced by the observed diffusion impedance in the low-frequency region (Figs S12 and S13). Additionally, the activation energy (E_a_) of Zn ions transference, calculated by the Arrhenius equation, further suggests the exceptional diffusion kinetics of Zn ions in the ZnCe electrode (Fig. 3h).

**Figure 4.**
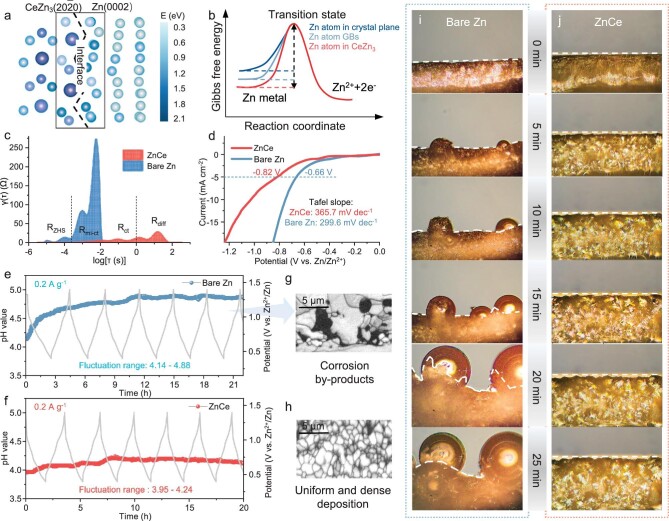
Corrosion and hydrogen evolution inhibition. (a) E_site_ of the Zn atom in Zn(0002) plane and at the interface between Zn(0002) and CeZn_3_(20${\mathrm{\overline{2}}}$0). (b) Variation curve of Gibbs free energy. (c) Evolution of EIS for bare Zn anode and URAL anode in Zn||NVO cells, along with the corresponding distribution of relaxation times (DRT) plot. (d) Hydrogen evolution curves of cells with bare Zn and URAL anode in 1 M NaSO_4_ electrolyte at 5 mV s^−1^. (e, f) pH value near the electrodes of the Zn||NVO cells with 3 M ZnSO_4_ electrolyte and corresponding *in-situ* GCD curves. (g, h) Partial SEM images of bare Zn and ZnCe electrodes after plating at 2 mAh cm^−2^. (i, j) *In-situ* optical microscopy images of the Zn plating process at 5 mA cm^−2^.

GBs have long been recognized as critical locales for side reactions, a scenario where finer grains translate to increased boundary areas, potentially amplifying the instances of these reactions. Importantly, this research highlights the novel observation that Ce incorporation at these GBs facilitates electron localization, a mechanism that serves to mitigate the reaction activity of Zn. When Ce atoms are introduced into GBs, they tend to segregate at the GBs due to their large atomic radius, leading to a reduction in GB energy and effectively suppressing corrosion reactions [30]. The E_site_ of Zn atoms at the interface between the CeZn_3_(20${\mathrm{\overline{2}}}$0) plane and Zn(0002) plane increases from 1.35 to 1.85 eV, predicting an elevation of the energy barrier for the reaction of ${\mathrm{Zn\ }} - {\mathrm{\ 2}}{{{\mathrm{e}}}^ - }{\mathrm{\ = \ Z}}{{{\mathrm{n}}}^{{\mathrm{2 + }}}}$ (Figs 3f, 4a and b). It is worth noting that the E_site_ of Ce is much higher than that of Zn, which is conducive to the stability of the alloy. Although Ce serves as an active metal element, its content remains almost stable after 1000 cycles in Zn cells, as evidenced by the energy-dispersive X-ray spectroscopy (EDS) elemental mapping in Fig. S14. The side reactions are closely related to the generation of Zn_4_SO_4_(OH)_6_·nH_2_O (ZHS) by-products [31]. To probe the accumulation of surface by-products arising from hydrogen evolution and corrosion reactions, electron probe microanalysis (EPMA) was carried out to examine the deposition morphology on the Zn electrodes. As depicted in Fig. S15, the surface of cycled bare Zn after 1000 cycles at 5 A g^−1^ exhibits irregular deposition, due to heterogeneous plating/stripping dynamics or corrosive interactions. EPMA mapping analysis of ZnCe electrode after cycling reveals a homogeneous dispersion of Zn (81.8%) with lower S content (0.3%) compared to the bare Zn electrode (2.9%), underscoring the superior corrosion resistance and the high reversibility of Zn plating/stripping. Furthermore, XRD testing was conducted on the cycled electrodes. Noticeable peaks are observed at the 8° and 17° positions on the bare Zn electrode, which are identified as the Zn_4_SO_4_(OH)_6_·nH_2_O by-product. In contrast, these peaks are scarcely observed in the XRD spectrum of the corresponding ZnCe electrode, indicating the significant role of ZnCe electrodes in suppressing corrosion (Fig. S16). Due to the overlap of several electrochemical processes between the observed electrochemical impedance spectroscopy (EIS) curves, a DRT analysis (Fig. 4c) was performed to resolve the electrochemical processes [32]. The peak of ${\mathrm{log}}[ {{\mathrm{\tau }}( {\mathrm{s}} )} ]{\mathrm{ = \ }} - {\mathrm{4}}$ is attributed to the adsorption and desorption of Zn^2+^ and associated with the charge transfer process of the ZHS (R_ZHS_) [33]. The pronounced peak of R_ZHS_ on the bare Zn electrode, compared to its almost absence on the ZnCe electrode, highlights the superior corrosion resistance of the ZnCe electrode. Linear sweep voltammetry (LSV) can reveal the electrochemical activity of the electrode by measuring the current response as a function of applied potential (Fig. 4d and Fig. S17). Under a rate of 5 mV s^−1^, the increase in current density for the ZnCe electrode is slower compared to the corresponding bare Zn electrode. By fitting the LSV plot to the Tafel equation, the Tafel slope was calculated to further evaluate the rate of hydrogen evolution reaction (HER) of the Zn anode. The Tafel slope for the Zn ion on the ZnCe electrode and the bare Zn electrode is 365.7 and 299.6 mV dec^−1^, respectively, indicating the inhibitory effect of the ZnCe electrode on HER. An *in-situ* pH monitoring device of electrolyte in Zn||NVO cells was utilized to evaluate the corrosion behavior of Zn electrodes (Fig. 4e and f). The pH value of ZnCe electrode maintains a stable weak acid environment, which could suppress corrosion reactions [34]. Notably, local SEM images in EBSD of the plated bare Zn and ZnCe electrodes were detected after argon ion polishing (Fig. 4g and h). For bare Zn, grey parts in images are identified as active Zn, whereas the black parts represent the unrecognizable phase regarded as corrosion by-products, revealing that corrosion reaction occurs at GBs and then extends along GBs. As a result, the corrosion reactions of Zn with electrolytes on ZnCe electrodes are substantially alleviated. We performed *in-situ* optical microscopy to monitor Zn deposition behavior. As the deposition time increases, Zn dendrites grow progressively with large bubbles on the bare Zn surface due to the severe HER (Fig. 4i). In sharp contrast, ZnCe electrodes maintain a dendrite-free and homogeneous morphology throughout the depositing process (Fig. 4j), further illustrating that it effectively suppresses dendrite growth and side reactions.

**Figure 5.**
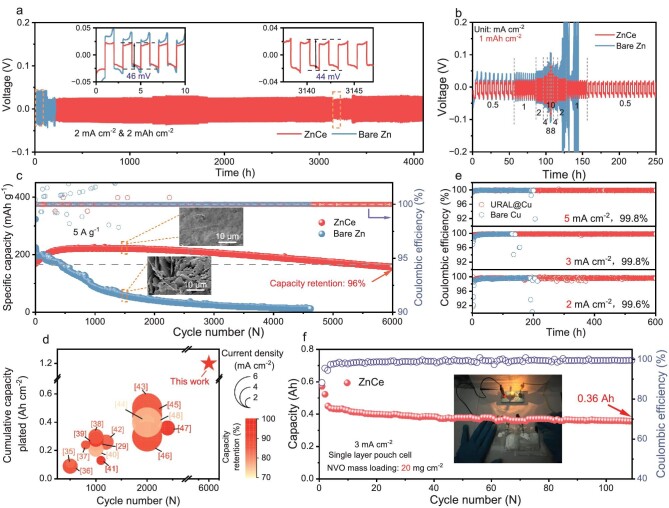
Electrochemical performances of cells with bare Zn and ZnCe electrodes. (a) Long-term galvanostatic cycling of Zn||Zn symmetric cells at 2 mA cm^−2^/2 mAh cm^−2^. (b) Rate performance of Zn||Zn symmetric cells at various current densities ranging from 0.5, 1, 2, 4, 8, 10 mA cm^−2^ and back to 0.5 mA cm^−2^ with a capacity of 1 mAh cm^−2^. (c) Cyclic stability of coin full cells with bare Zn anode and ZnCe anode. (d) Comprehensive comparison of cycling performances with other Zn||NVO coin cells previously reported. (e) CEs of the Zn plating/stripping process in Cu||Zn cells using URAL@Cu electrode and bare Cu electrode. (f) Cycling performance of the single-layer ZnCe||NVO pouch cell, after activating for 2 cycles at 1 mA cm^‒2^ (inset shows the corresponding light bulb experiment).

The Zn stripping/plating tests were performed in galvanostatic cycling of Zn||Zn symmetric cells under a current density of 2 mA cm^−2^ with a capacity of 2 mAh cm^−2^ (Fig. 5a). The ZnCe electrodes exhibit remarkable cyclic reversibility, which can operate over 4000 h with a reduced voltage hysteresis compared to bare Zn. Such high stability and long cycling life are superior to most of the previously reported Zn||Zn symmetric cells (Fig. S18). Meanwhile, we further assessed the morphology evolution of the Zn electrodes after 100 cycles by atomic force microscope (AFM) (Fig. S19). The cycled bare Zn presents a coarse surface, with a height intercept of 5.3 μm. By contrast, the cycled ZnCe surface is relatively smooth and dense, with a low-altitude intercept of 1.6 μm. These results are attributed to the large massive dendrite and corrosion by-products observed on bare Zn electrodes from the *ex-situ* SEM images (Fig. S20), while the ZnCe electrodes remain flat and compact without dendrites. To evaluate the universal applicability of the outstanding performance of ZnCe electrodes, we examined the cyclic stability of symmetrical cells at higher (5 mA cm^−2^) and lower (1 mA cm^−2^) current densities. The performance of symmetrical cells equipped with ZnCe electrodes surpasses that of bare Zn at both higher and lower current densities (Figs S21 and S22). In addition, Fig. 5b displays the rate performances for Zn symmetric cells under varied current densities from 0.5 to 10 mA cm^−2^, and then back to 0.5 mA cm^−2^ at a fixed capacity of 1 mAh cm^−2^. Indeed, in comparison to Zn||Zn symmetrical cells, the ZnCe||ZnCe symmetrical cells demonstrate reduced voltage hysteresis (Fig. S23), as supported by the findings from EIS. As illustrated in Fig. S24, the charge transfer impedance (R_ct_) at the ZnCe electrode interface is lower, possibly due to enhanced Zn^2+^ transport kinetics resulting from increased GBs. Moreover, after 10, 20, and 30 cycles, the R_ct_ of the bare Zn electrode notably decreases from 473 to 106, 52 Ω, and subsequently exhibits fluctuations. In contrast, the R_ct_ of the ZnCe electrode remains relatively stable. This observation highlights the superior cycling reversibility of the ZnCe electrode.

Furthermore, NVO was synthesized using traditional hydrothermal methods, with its morphology shown in Fig. S25. The CV curves of the assembled ZnCe||NVO full cells present closer overlapping redox peaks, suggesting faster electrochemical dynamics in comparison to bare Zn (Fig. S26). Notably, at a current density of 5 A g^−1^, the ZnCe||NVO cell exhibits remarkable long-term stability, maintaining over 96% capacity retention for over 6000 cycles (Fig. 5c). The corresponding charge/discharge curves are shown in Fig. S27. Such a high cumulative capacity and long lifespan have an advantage over most of the recently reported Zn||NVO cells due to the high reversibility of ZnCe anode (Fig. 5d) [35–48]. Correspondingly, the capacity of Zn||NVO cells assembled with bare Zn anode quickly decays around 500 cycles, indicative of cell failure. The inset illustrates that the short lifespan of cells with bare Zn is due to the generation of severe Zn dendrites and loose corrosion by-products, but the ZnCe electrodes maintain a dense, uniform surface morphology after cycling, indicating the dendrite-free plating/stripping process. It is noteworthy that, even under low current densities of 1 and 2 A g^−1^, the ZnCe||NVO full cells also demonstrate significantly improved cycling stability compared to the bare Zn||NVO full cells (Figs S28 and S29). In addition, full cells with ZnCe electrodes exhibit superior rate performance, with harvest capacities of 344, 323, 291, 215, and 136 mAh g^−1^ at 0.5, 1, 2, 5, and 10 A g^−1^, respectively. In contrast, cells with bare Zn electrodes show capacities of 343, 310, 244, 115, and 65 mAh g^−1^ at equivalent current densities, markedly lower than the full cells with ZnCe electrodes (Fig. S30). This disparity may arise from the ZnCe electrode's advantages in enhancing Zn^2+^ transport kinetics. Despite a larger current density, the reversibility of the Zn anode becomes worse due to rapid Zn dendrite growth and severe side reactions. However, by testing the reversibility of Zn stripping/plating in Cu||Zn cells at different current densities, it is found that the ZnCe electrode exhibits enhanced reversibility under the effect of URAL (Fig. 5e). Encouragingly, a large-scale pouch cell was successfully assembled by ZnCe anode (10 cm × 10 cm in size) and NVO cathode with a high loading of 20 mg cm^−2^ (Fig. 5f and Table S2). It delivers a slight capacity drop from the initial 0.43 to 0.36 Ah after 110 cycles at 3 mA cm^−2^, and can operate a bulb.

## CONCLUSION

In summary, a strategy for refining the electrodeposited layer grains is proposed in order to improve the electrochemical performance of Zn anodes. Both experimental and theoretical findings reveal grain refinement could evenly compact the deposition layer and inhibit dendrite growth. The uniform distribution of particles on the electrode surface alters the electric field distribution at the electrode/electrolyte interface, and the abundance of GBs further improves Zn-ion transport and deposition kinetics. Additionally, *in-situ* pH measurements, EBSD analysis, *in-situ* optical microscopy, and E_site_ calculations systematically demonstrate the inhibition of corrosion and hydrogen evolution by the addition of Ce in electrodes. Benefiting from these merits, integrated URAL@Cu||Zn cells exhibit a high average CE of 99.8% at a current density of 3 mA cm^−2^, and the symmetric ZnCe||ZnCe cell presents superior cycling performance (over 4000 h) at 2 mA cm^−2^ with a capacity of 2 mAh cm^−2^. It is worth noting that the ZnCe||NVO single-layer pouch cell with a high-loading cathode (20 mg cm^−2^) shows an impressive capacity of ∼0.4 Ah and runs over 110 cycles. This work sheds light on a fundamental explanation of Zn metal nucleation and growth, presenting a promising manipulation method for grain regulation to optimize metal anodes.

## Supplementary Material

nwae205_Supplemental_File
