# Supplementary material for: Spontaneous grain refinement effect of rare earth zinc alloy anodes enables stable zinc batteries
Source: Natl Sci Rev. 2024 Jun 13;11(7):nwae205. doi: 10.1093/nsr/nwae205 (PMC11275459; doi:10.1093/nsr/nwae205)
Supplement: nwae205_Supplemental_File [file nwae205_supplemental_file.pdf]

Supplementary Information for

Spontaneous Grain Refinement Effect of Rare Earth Zinc Alloy Anodes Enables Stable Zinc Batteries

*Manjing Chen[1], Yuxiang Gong[1], Yunxiang Zhao[2], Yexin Song[1], Yan Tang[1], Zhiyuan Zeng[3], Shuquan Liang[1], Peng Zhou[4], Bingan Lu[5], Xiaotan Zhang[*1,6,7] and Jiang Zhou[*1]*

[1]School of Materials Science & Engineering, Hunan Provincial Key Laboratory of Electronic Packaging and Advanced Functional Materials, Central South University, Changsha 410083, China;[2]Institute of Materials Research, Tsinghua Shenzhen International Graduate School, Tsinghua University, Shenzhen 518055, China;[3]Department of Materials Science and Engineering, City University of Hong Kong, Hong Kong 999077, China; [4] Hunan Provincial Key Defense Laboratory of High Temperature Wear-Resisting Materials and Preparation Technology, Hunan University of Science and Technology, Xiangtan 411201, China;[5]School of Physics and Electronics, Hunan University, Changsha 410082, China;[6]School of Chemistry and Materials Science, University of Science and Technology of China, Hefei 230026, China; [7]Suzhou Institute for Advanced Research, University of Science and Technology of China, Suzhou 215123, China.

[*]Corresponding author. E-mail: zhangxiaotan@ustc.edu.cn; zhou_jiang@csu.edu.cn

Methods

Fabrication of ZnCe electrodes

70 g Zn sulfate heptahydrate ($ZnSO_4 \cdot 7\ H_2O$), 2.137 g Cerous sulfate ($Ce_2(SO_4)_3 \cdot 8\ H_2O$) and 1.176 g $C_6H_6Na_2O_7$ were dissolved in the 200 ml water with continuous stirring until a transparent solution was obtained to form the electroplating solution. A self-made electrochemical cell was used to electrodeposition Zn-Ce alloy on Zn foil (ZnCe electrode) with another Zn foil as the counter electrode at 3 mA cm^{-2} for 18 min. The obtained ZnCe electrode could be visually seen as colored. By adjusting the preparation conditions of the electrodeposition process, such as varying the salt concentration ratio, current density, and electrodeposition time, different outcomes can be achieved.

Synthesis of $NH_4V_4O_{10}$ cathode material

In a typical process, 1.17 g of NH_4VO_3 was dissolved in 90 ml of water. After being magnetically stirred for 1 h at 80 °C, 3.4038 g of $H_2C_2O_4 \cdot 2\ H_2O$ was added and stirred for a while. Then, the solution was transferred to a Teflon-lined autoclave and heated at 140 °C for 48 h. Finally, the above solution was washed with deionized water and ethanol for several times and dried at 80 °C for 12 h during a vacuum atmosphere to obtain the $NH_4V_4O_{10}$ product.

Materials characterization

The electron backscattered diffraction (EBSD) measurements were recorded on an Oxford Symmetry 2 apparatus. Scanning electron microscopy (SEM) images were collected on a field emission scanning electron microscope (FESEM, MIRA3 LMH, TESCAN, 15 kV) combined with an energy dispersive X-ray spectrometer (EDS, Oxford XMAX20). Crystal structures and compositions of the electrode were carried out on powder X-ray diffraction (XRD, Rigaku Mini-Flex 600 diffractometer) operated with Cu Kα radiation (λ = 0.15418 nm) and further confirmed by transmission electron microscope (TEM, JEOL JEM-F200). X-ray photoelectron spectroscopy (XPS) was conducted on a thermos ESCALAB 250 photoelectron spectrometer using monochromatic Al Kα radiation under vacuum at hv = 1486.6 eV. Confocal laser scanning microscope (CLSM, LEXT OLS4100) was used to observe

the initial morphology of the electrodes. The wettability of the electrodes was measured by a contact angle measuring device (Lauda Scientific LSA100, Germany). The in-situ optical microscope studies were conducted using an LW750LJT translative optical microscope. Electrode flatness after cycling was measured by an atomic force microscope (Bruker, DIMENSION ICON).

Electrochemical measure

Electrochemical characterization of symmetrical cells was conducted using 2025-type coin-cells with 3 M $ZnSO_4$ as the electrolyte. Galvanostatic discharge-charge profiles were collected using LAND-CT2001A. Linear sweep voltammetry (LSV), Tafel, electrochemical impedance spectroscopy (EIS), and cyclic voltammetry (CV) tests were carried out on an electrochemical workstation (CHI660E, Chenhua, China). Chronoamperometry (CA) curves were acquired using a three-electrode cell with Zn foil as the working electrode, platinum foil as the counter electrode, and Ag/AgCl as the reference electrode, respectively. The in-situ pH of the 3 M $ZnSO_4$ electrolyte near the electrode in the cuvette was tested using desktop pH tester (JENCO 6173).

Density functional theory (DFT) calculation

DFT calculations were performed using the Vienna Ab-initio Simulation Package (VASP). The projector augmented wave method was used to describe the ion-electron interactions. Generalized gradient approximation (GGA) in the scheme of Perdew-Burke-Ernzerhof (PBE) was used to describe electron exchange and correlation interactions. A kinetic energy cutoff of 480 eV was applied for the plane wave expansion of the valence electron wave functions. The atomic relaxation was stopped when the total energy tolerance had converged to 10^{-5} eV, and the changes of the force on atoms were less than 0.05 eV $Å^{-1}$. The charge density difference was calculated by subtracting the charge density of the individual components from that of the system.

COMSOL simulation

A simplified three-dimensional electric field model of the Zn electrode surface based on COMSOL was developed to clarify the role of the ZnCe electrode in achieving a uniform electric field distribution

and inhibiting Zn dendrite growth. Specifically, electrochemical simulations were conducted using the COMSOL Multiphysics framework with the tertiary current distribution and Nernst-Planck interface.

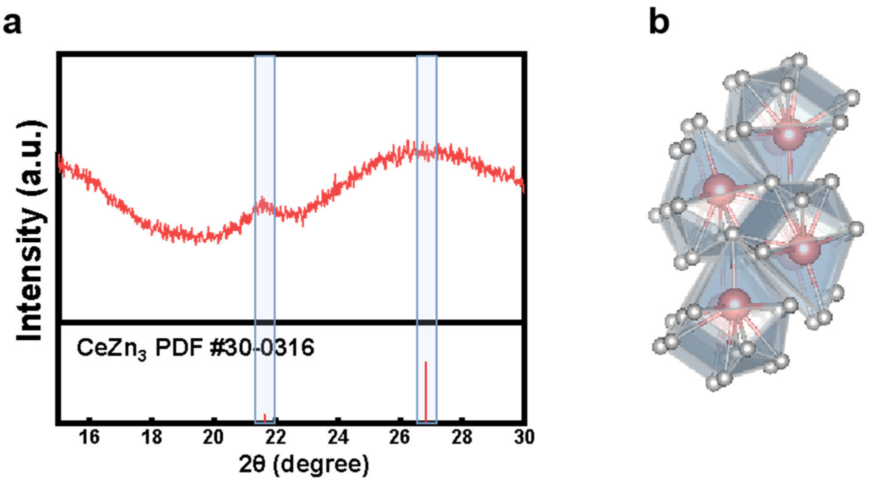

Fig. S1. (a) XRD patterns of ZnCe electrode. (b) Crystal structure of $CeZn_3$.

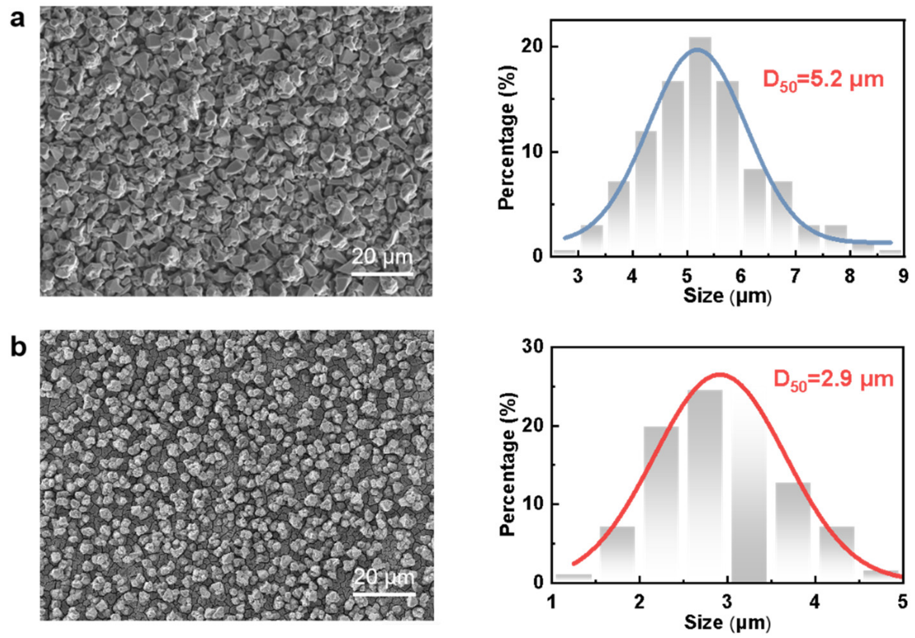

Fig. S2 Electrode morphology and surface particle size distribution map prepared by electrodeposition at (a) 6 mA cm^{-2} and (b) 3 mA cm^{-2}.

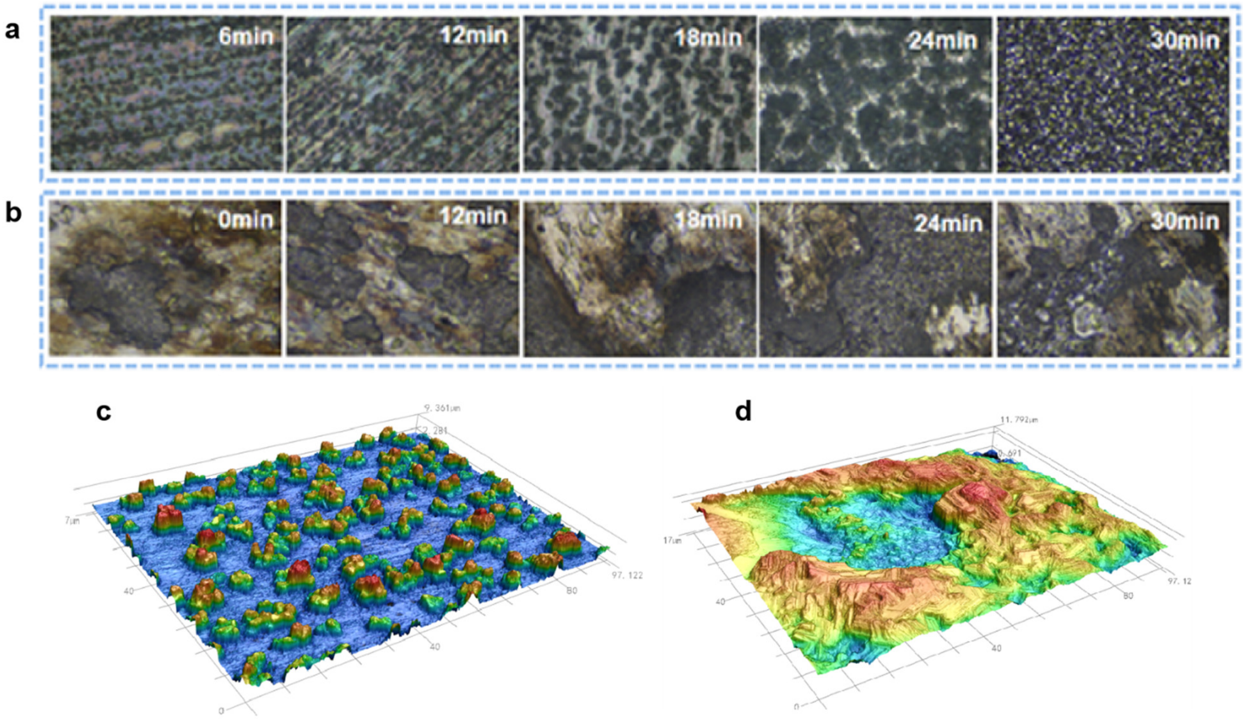

Fig. S3. The morphology of electrode obtained by electrodeposition at different time in (a) the prepared plating solution and (b) 70 g L^{-1} $ZnSO_4$ solution. (c, d) Corresponding laser scanning confocal microscope images of the electrodes obtained by electrodeposition for 18 min.

Based on the electrode morphology described above, we have chosen suitable electrodeposition conditions to distribute uniform particles on the electrode surface, namely a current density of 3 mA cm^{-2} and an electrodeposition time of 18 min.

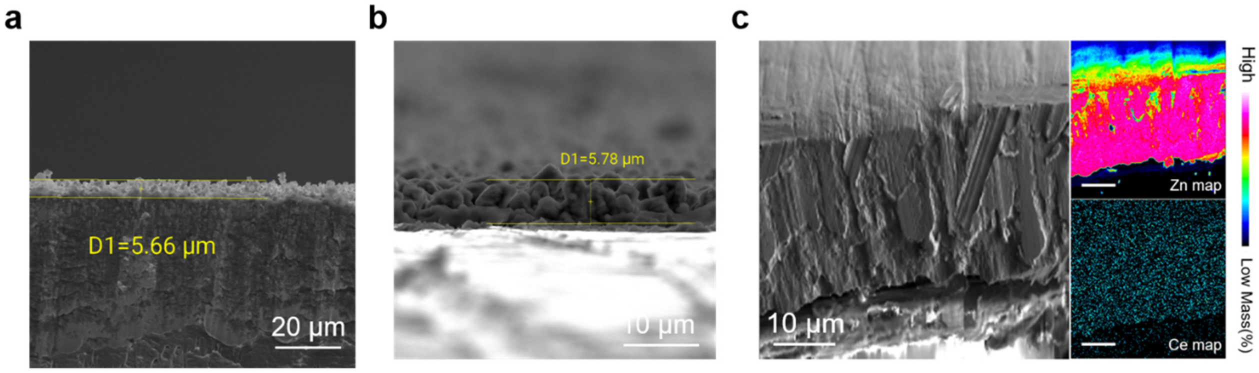

Fig. S4. (a, b) The cross-section SEM images of ZnCe electrode. (c) The uniformity of element distribution was determined by EPMA.

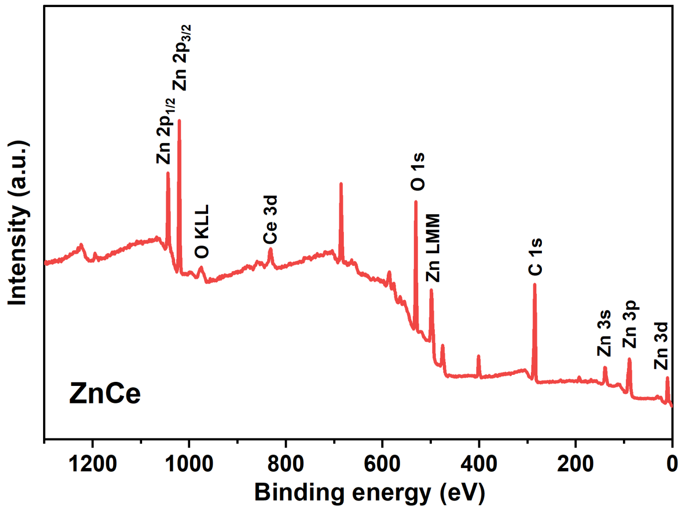

Fig. S5. XPS spectra of the ZnCe electrode.

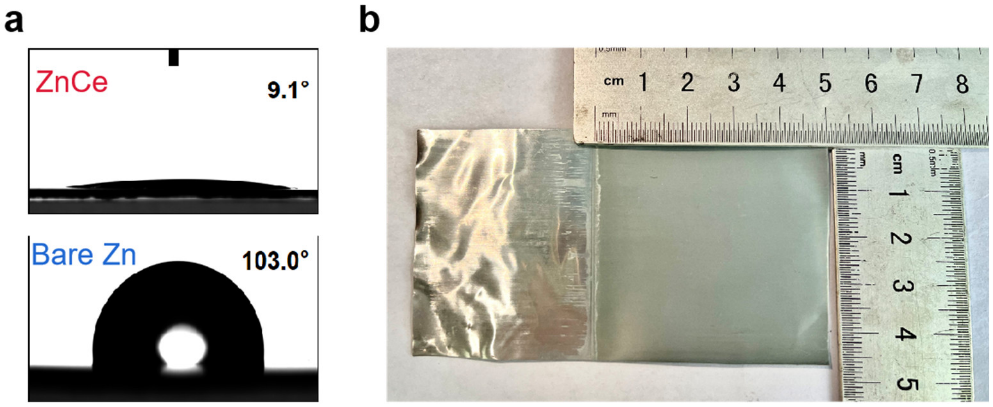

Fig. S6. (a) Static contact angle measurements of ZnCe electrode and bare Zn in 3 M $ZnSO_4$. (b) Digital image of prepared ZnCe electrode.

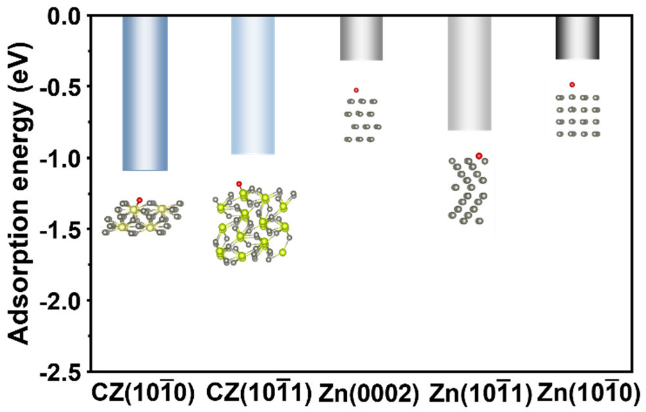

Fig. S7. Modeling and the corresponding calculated adsorption energy of a Zn atom on $CeZn_3(10\bar{1}0)$, $CeZn_3(10\bar{1}1)$, Zn(0002), Zn(10$\bar{1}$1) and Zn(10$\bar{1}$0), respectively.

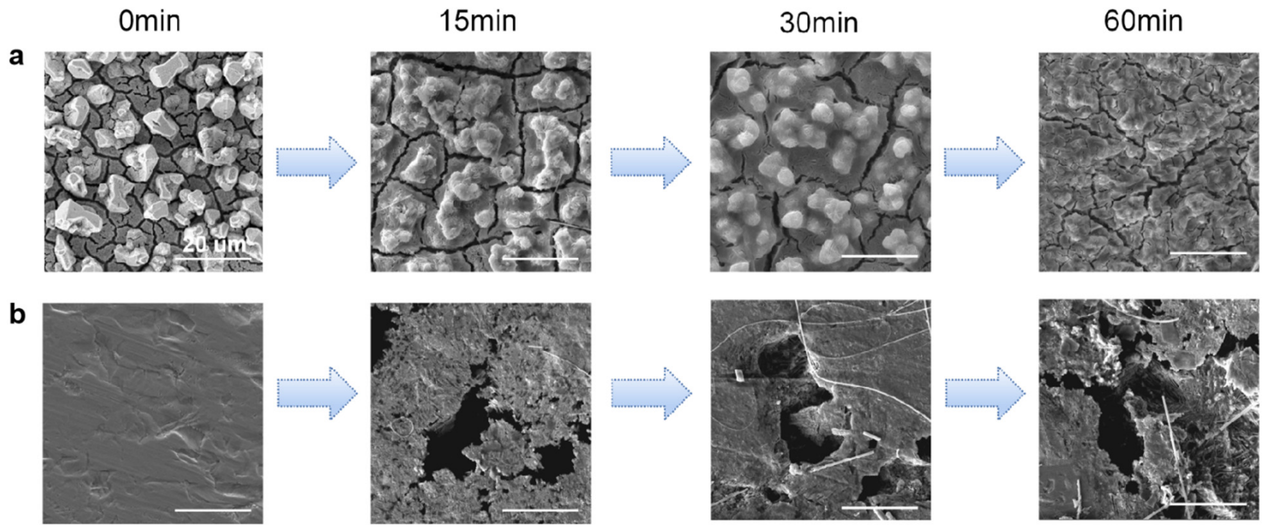

Fig. S8. Ex-situ SEM images of (a) ZnCe and (b) bare Zn electrodes after stripping at a current density of 2 mA cm^{-2}.

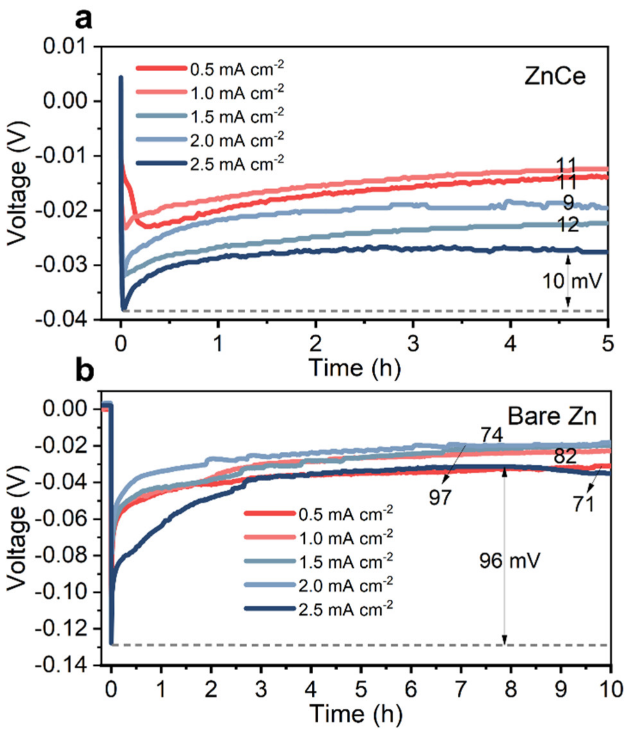

Fig. S9. Galvanostatic nucleation overpotential (NOP) of Zn deposition based on (a) ZnCe and (b) bare Zn electrodes for 3 M $ZnSO_4$ electrolyte at different current densities.

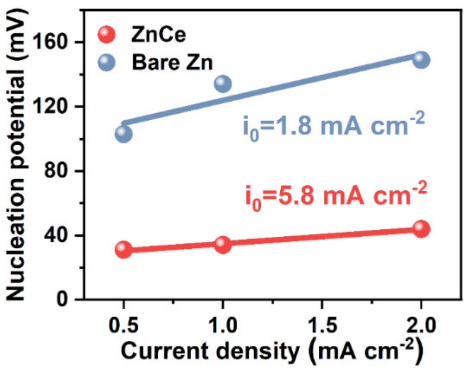

Fig. S10. Exchange current density at different current densities.

To evaluate the deposition kinetics in more detail, we calculated the exchange current density associated with Zn deposition, the equation can be expressed as follow:

$$i = i_0 \frac{F}{RT}\frac{\eta}{2} \quad (1)$$

where i is the current density, i_0 is the exchange current density, F is the Faraday constant, R is the gas constant, T is the absolute temperature, and η is the total overpotential. As shown in Fig. S10, the ZnCe electrode has a higher exchange current density value.

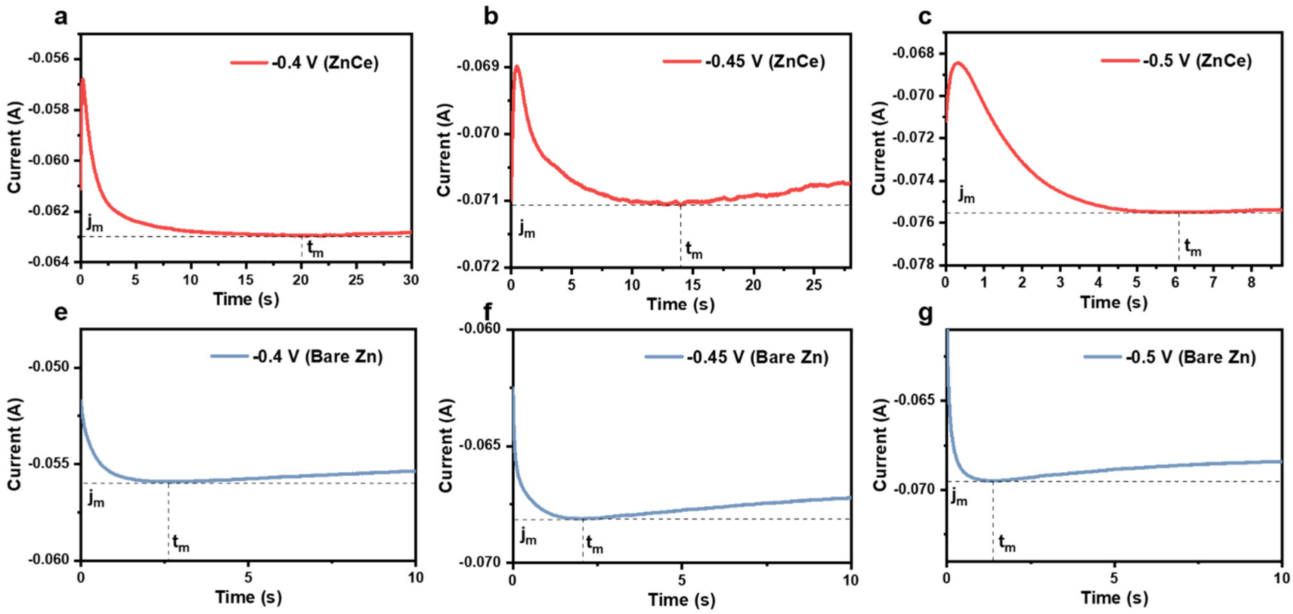

Fig. S11. Current-time transients obtained at a given potential.

The nucleation model of bare Zn and ZnCe electrodes in 3 M $ZnSO_4$ electrolyte were studied by chronoamperometry to understand the cause of grain refinement in Zn deposits. According to the Scharifker model for electrochemical nucleation, nucleation occurs in two forms, instantaneous and progressive. This theory describes the transient behavior of static current for three-dimensional multiplex nuclei with diffusion-controlled growth, and the corresponding expressions are as follows:

$$\text{Instantaneous: } (j/j_m)^2 = 1.9542\,(t/t_m)^{-1}\{1 - exp[1.2564\,(t/t_m)]\}^2 \quad (2)$$

$$\text{Progressive: } (j/j_m)^2 = 1.2254\,(t/t_m)^{-1}\{1 - exp[-2.3367\,(t/t_m)^2]\}^2 \quad (3)$$

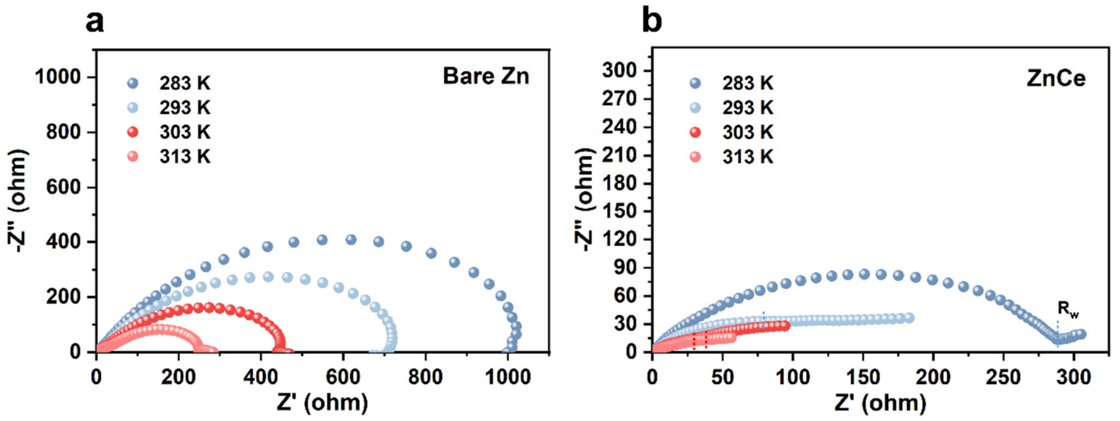

Fig. S12. Impedance of the assembled (a) bare Zn||Zn and (b) ZnCe||ZnCe symmetric cells at different temperatures.

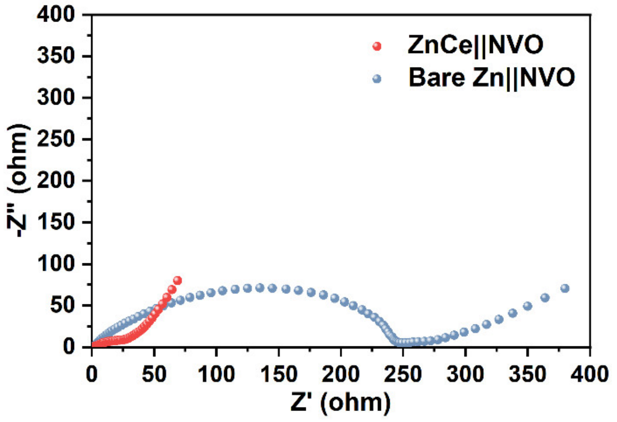

Fig. S13. Fitted Nyquist plots of the full cells in the initial state.

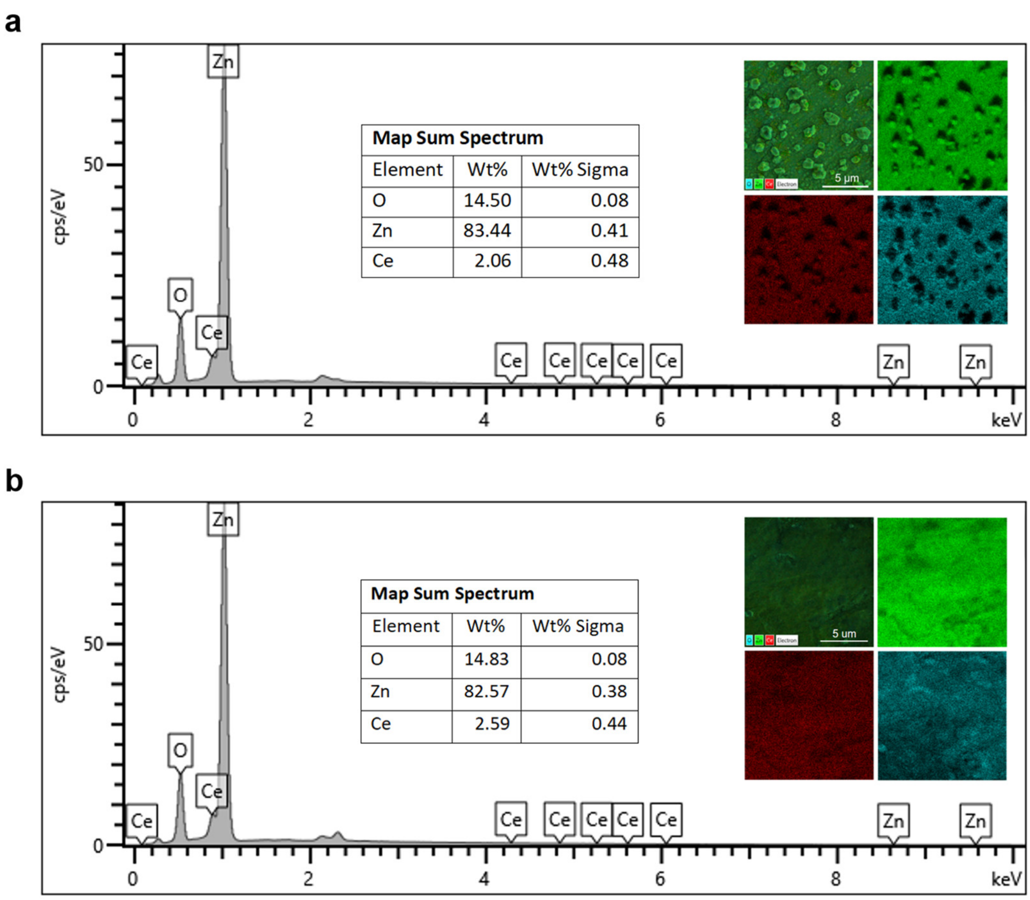

Fig. S14. The EDS elemental mapping of the ZnCe electrode (a) before and (b) after 1000 cycles.

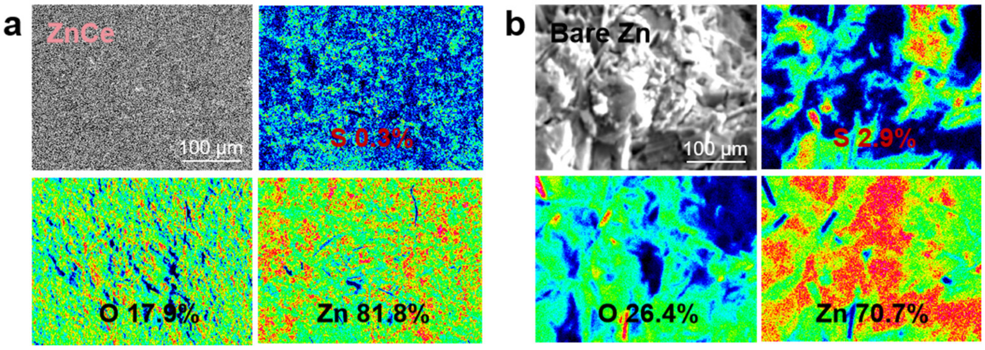

Fig. S15. EPMA images of (a) ZnCe and (b) bare Zn electrodes subjected to 1000 cycles at 5 A g^{-1}.

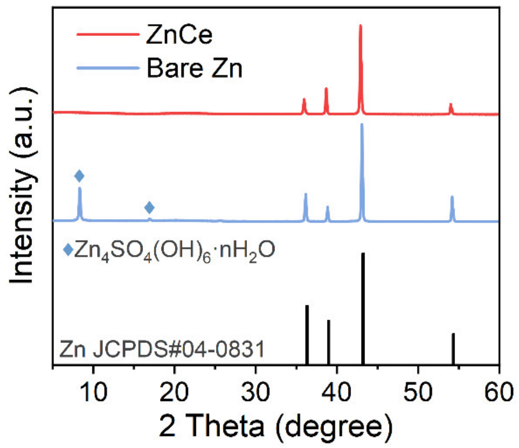

Fig. S16. XRD spectrum of ZnCe and bare Zn electrodes subjected to 1000 cycles at 5 A g^{-1}.

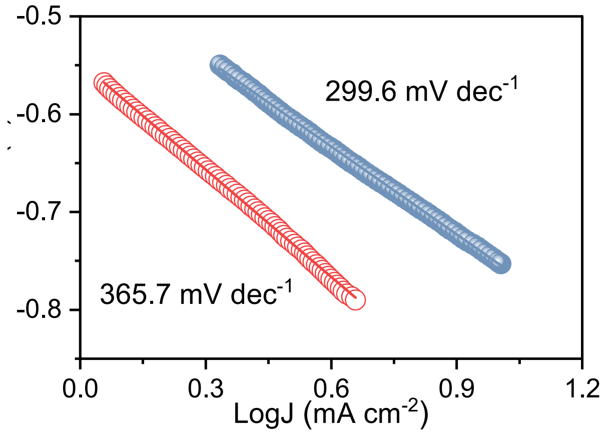

Fig. S17. Calculated Tafel slopes of hydrogen evolution curves at 5 mV s^{-1}.

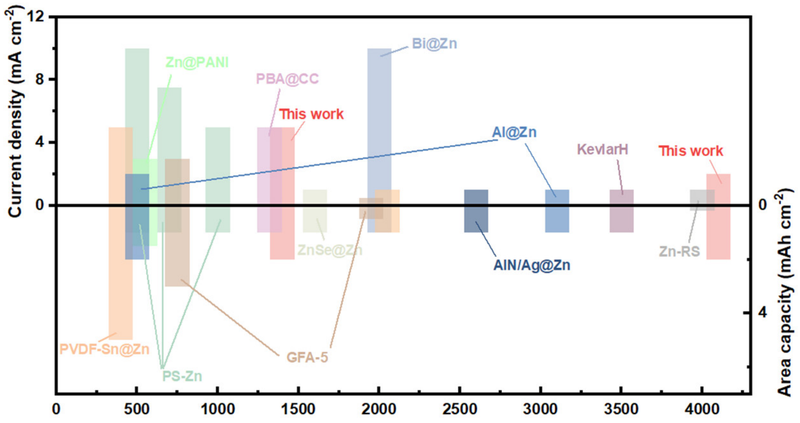

Fig. S18. Cyclic reversibility comparison of symmetric cells in the recent report [1-11].

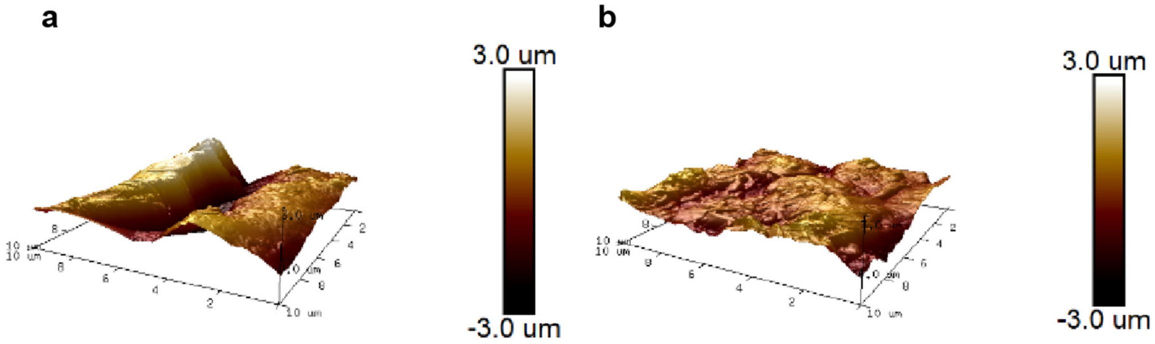

Fig. S19. AFM images of (a) bare Zn and (b) ZnCe electrodes after 100 cycles at 2 mA cm^{-2}/2 mAh cm^{-2}.

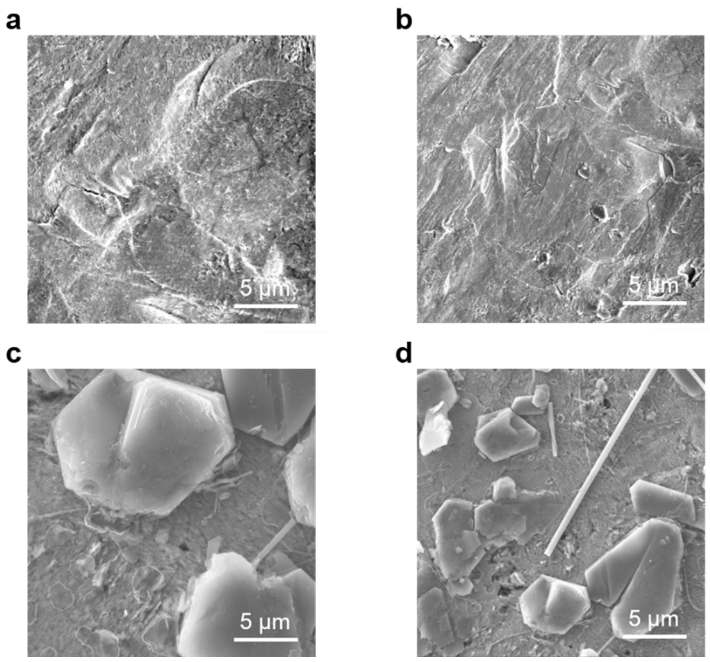

Fig. S20. Top-view SEM images of (a, b) ZnCe and (c, d) bare Zn electrodes after cycling for 200 h at 2 mA cm^{-2} and 2 mAh cm^{-2}.

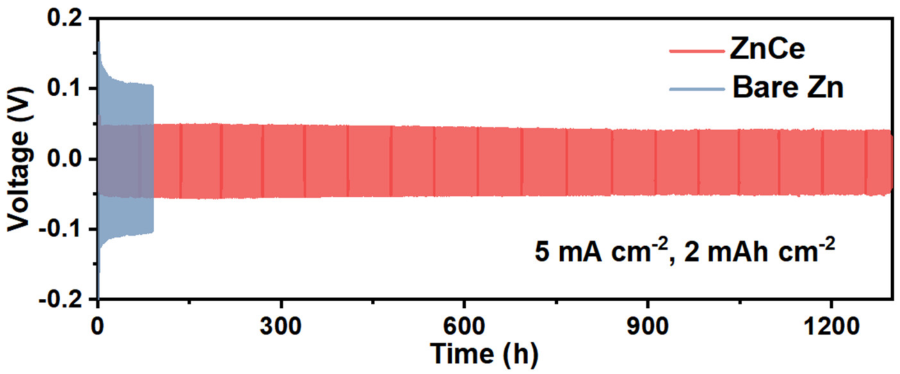

Fig. S21. Voltage profiles of symmetric cells at 5 mA cm^{-2}/2 mAh cm^{-2}.

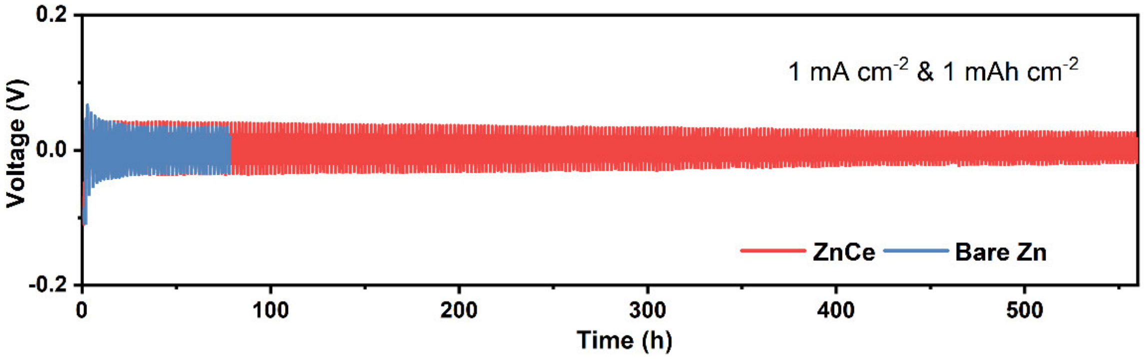

Fig. S22. Long-term galvanostatic cycling of Zn||Zn symmetric cells at 1 mA cm^{-2} and 1 mAh cm^{-2}.

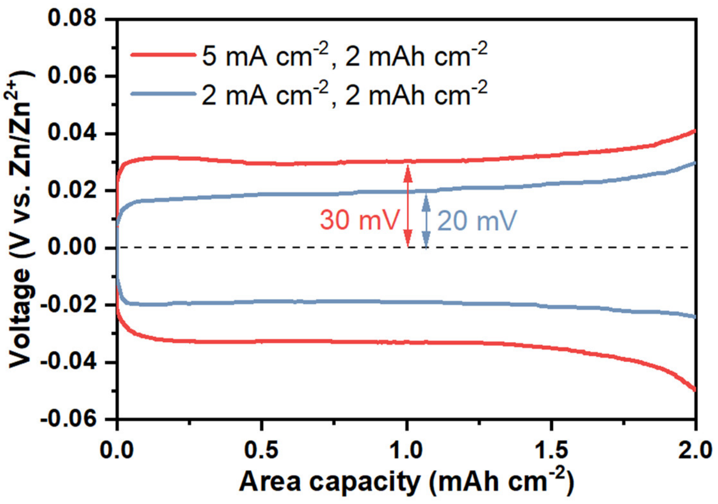

Fig. S23. Voltage profiles of ZnCe||ZnCe symmetric cells after 10 cycles at different current densities.

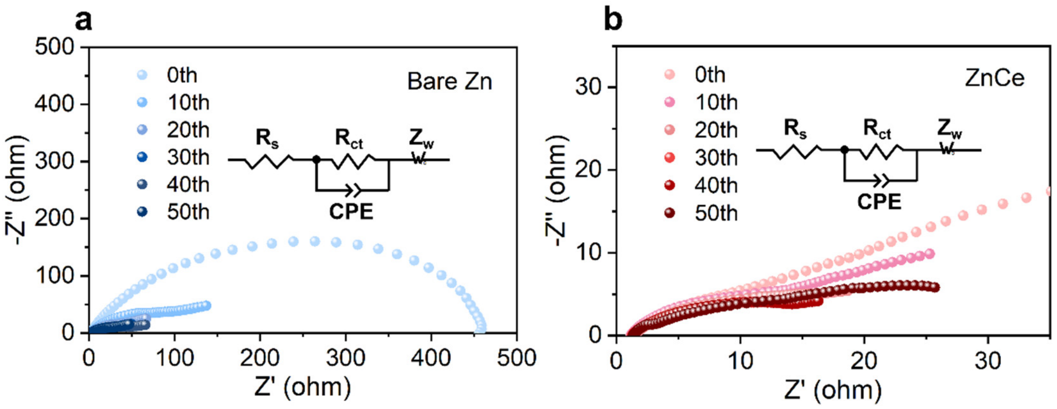

Fig. S24. Nyquist plots of symmetric cells using (a) bare Zn and (b) ZnCe electrodes cycling for different cycles at 2 mA cm^{-2} and 2 mAh cm^{-2}.

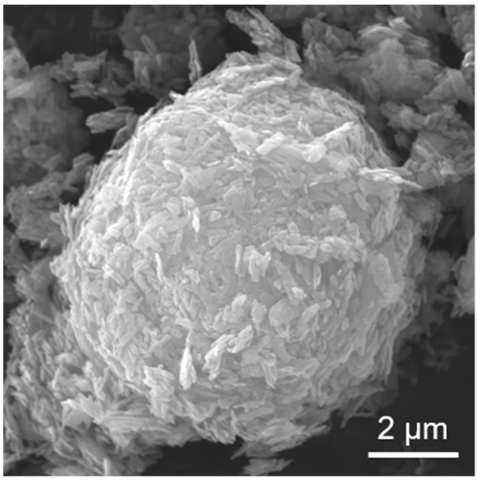

Fig. S25. SEM image of $NH_4V_4O_{10}$ cathode.

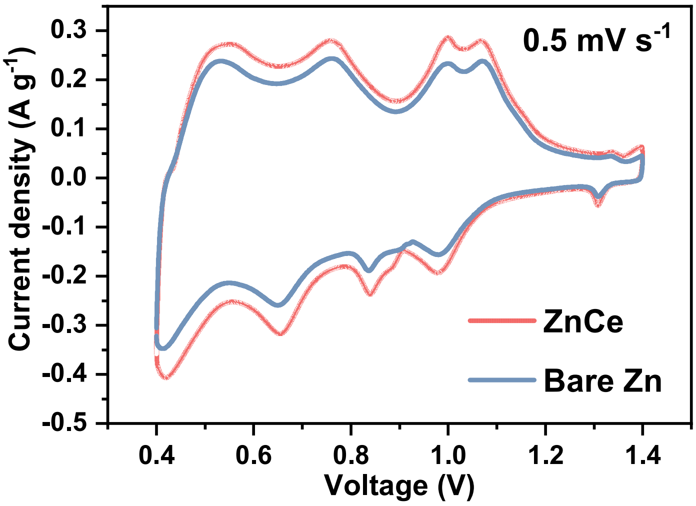

Fig. S26. CV curves of full cells at 0.5 mV s^{-1}.

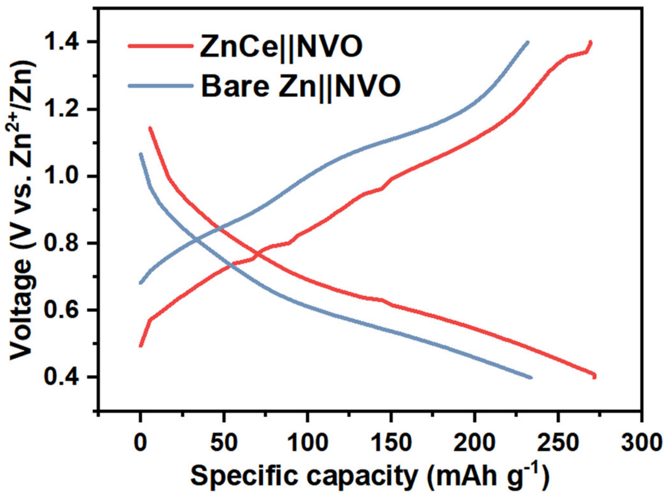

Fig. S27. Voltage profiles for the first cycle of full cells at 5 A g^{-1}.

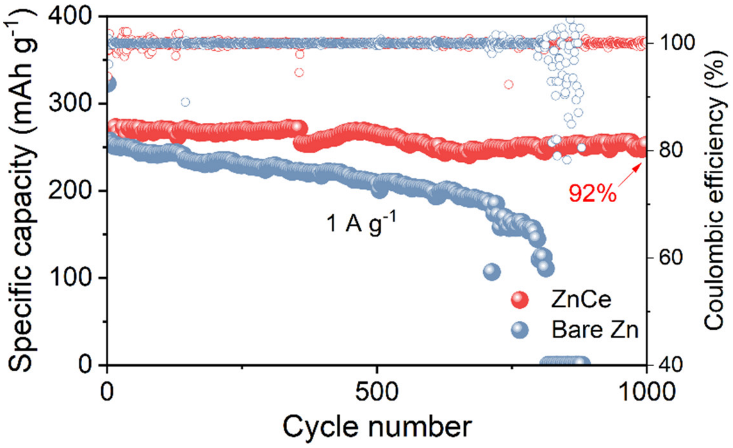

Fig. S28. The long-term performance of ZnCe||NVO and bare Zn||NVO full cells at 1 A g^{-1}.

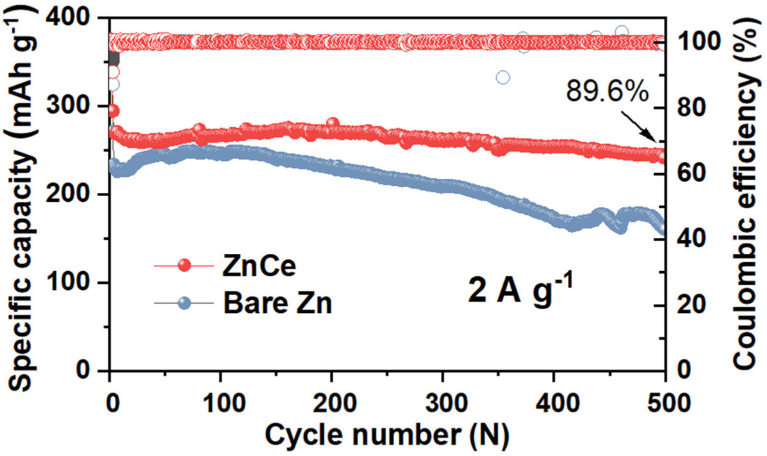

Fig. S29. The long-term performance of ZnCe||NVO and bare Zn||NVO full cells at 2 A g^{-1}.

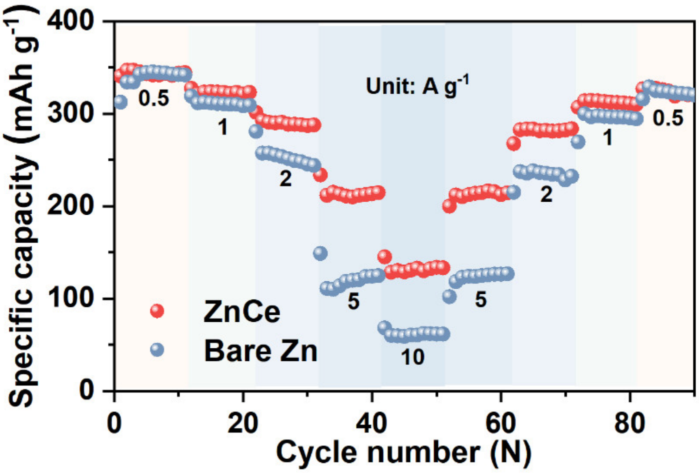

Fig. S30. Rate performance of full cells from 0.5 to 10 A g^{-1}.

Table S1. The inductively coupled plasma optical emission spectrometer (ICP-OES) data of ZnCe electrodes.

Element	Ce	Zn
Mass fraction (%)	0.83	Bal.
Atomic ratio (%)	0.39	Bal.

Table S2. The cycling performance of the pouch cells in recent demonstrations compared to our work.

Type	Cell configuration	Current density (mA cm^{-2})	Capacity retention ratio (%)	Cycle number	Capacity (Ah)	Ref.
electrolyte	ZIB/PAH-PCH//I_2	-	99	100	0.03	[12]
anode	ZnHCF//VOH	2	93	100	0.06	[13]
electrolyte	ZSO-CoSA/C//V_2O_5	0.2	67	180	0.005	[14]
electrolyte	SPS10//VOH	1	77	200	0.02	[15]
electrolyte	BMIm//$NH_4V_4O_{10}$	-	79	240	0.02	[16]
anode	Zn@ZBO//MnO_2	-	65	100	0.77	[17]
anode	Zn/GP-HVO_d//MnO_2	1.25	89	50	0.71	[18]
**anode**	**ZnCe//$NH_4V_4O_{10}$**	**3**	**83**	**110**	**~0.4**	**This work**

References

1. Cao QH, Gao Y and Pu J *et al.* Gradient design of imprinted anode for stable Zn-ion batteries. *Nat Commun* 2023; **14**: 641.
2. Zou PC, Nykypanchuk D and Doerk G *et al.* Hydrophobic molecule monolayer brush-tethered zinc anodes for aqueous zinc batteries. *ACS Appl Mater* 2021; **13**: 60092-60098.
3. Li B, Liu SD and Geng YF *et al.* Achieving stable zinc metal anode via polyaniline interface regulation of Zn ion flux and desolvation. *Adv Funct Mater* 2024; **34**: 2214033.
4. Deng D, Fu K and Yu RH *et al.* Ion tunnel matrix initiated oriented attachment for highly utilized Zn anodes. *Adv Mater* 2023; **35**: 2302353.
5. Liang GJ, Zhu JX and Yan BX *et al.* Gradient fluorinated alloy to enable highly reversible Zn-metal anode chemistry. *Energy Environ Sci* 2023; **15**: 1086-1096.
6. Zhang L, Zhang B and Zhang T *et al.* Eliminating dendrites and side reactions via a multifunctional ZnSe protective layer toward advanced aqueous Zn metal batteries. *Adv Funct Mater* 2021; **31**: 2100186.
7. Zhao RZ, Dong XS and Liang P *et al.* Prioritizing hetero-metallic interfaces via thermodynamics inertia and kinetics zincophilia metrics for tough Zn-based aqueous batteries. *Adv Mater* 2023; **35**: 2209288.
8. Zheng JX, Huang ZH and Zeng Y *et al.* Electrostatic shielding regulation of magnetron sputtered Al-based alloy protective coatings enables highly reversible zinc anodes. *Nano Lett* 2022; **22**: 1017-1023.
9. Zheng JX, Wu YC and Xie HX *et al.* In situ alloying sites anchored on an amorphous aluminum nitride matrix for crystallographic reorientation of zinc deposits. *ACS Nano* 2022; **17**: 337-345.
10. Yang Y, Hua HM and Meng WW *et al.* Diminishing space-charge layer effect of zinc anodes by an anion-immobilized electrolyte membrane. *ACS Energy Lett* 2023; **8**: 1959-1968.
11. Wei SQ, Qi ZH and Xia YJ *et al.* Monolayer thiol engineered covalent interface toward stable zinc metal anode. *ACS Nano* 2022; **16**: 21152-21162.
12. Yang JL, Yu JW and Chen LY *et al.* Hetero-polyionic hydrogels enable dendrites-free aqueous Zn-I_2 batteries with fast kinetics. *Adv Mater* 2023; **35**: 2306530.
13. Liu MY, Yuan WT and Ma GQ *et al.* In-situ integration of a hydrophobic and fast-Zn^{2+}-

conductive inorganic interphase to stabilize Zn metal anodes. *Angew Chem Int Ed* 2023; **62**: e202304444.

14. Fan WJ, Li P and Chen JW *et al.* Atomic zincophilic sites regulating microspace electric fields for dendrite-free zinc anode. *Adv Mater* 2024; **36**: 202307219.
15. Lin YX, Mai ZX and Liang HK *et al.* Dendrite-free Zn anode enabled by anionic surfactant-induced horizontal growth for highly-stable aqueous Zn-ion pouch cells. *Energy Environ Sci* 2023; **16**: 687-697.
16. Zhang HW, Zhong Y and Li JB *et al.* Inducing the preferential growth of Zn (002) plane for long cycle aqueous Zn-ion batteries. *Adv Energy Mater* 2023; **13**: 2203254.
17. Wang DD, Liu HX and Lv D *et al.* Rational screening of artificial solid electrolyte interphases on Zn for ultrahigh-rate and long-life aqueous batteries. *Adv Mater* 2023; **35**: 2207908.
18. Huang JT, Liang HP and Tang Y *et al.* In situ induced coordination between a "desiccant" interphase and oxygen-deficient navajoite towards highly efficient zinc ion storage. *Adv Energy Mater* 2022; **12**: 2201434.
